# Chronic Stress A Potential Suspect Zero of Atherosclerosis: A Systematic Review

**DOI:** 10.3389/fcvm.2021.738654

**Published:** 2021-12-20

**Authors:** Ling-bing Meng, Yuan-meng Zhang, Yue Luo, Tao Gong, De-ping Liu

**Affiliations:** ^1^Department of Cardiology, National Center of Gerontology, Institute of Geriatric Medicine, Beijing Hospital, Chinese Academy of Medical Sciences, Beijing, China; ^2^Graduate School of Peking Union Medical College and Chinese Academy of Medical Sciences, Beijing, China; ^3^Department of Internal Medicine, The Third Medical Centre of Chinese People's Liberation Army (PLA) General Hospital, The Training Site for Postgraduate of Jinzhou Medical University, Beijing, China; ^4^Department of Respiratory, The First Affiliated Hospital of Jinzhou Medical University, Jinzhou, China; ^5^Department of Neurology, National Center of Gerontology, National Center of Gerontology, Institute of Geriatric Medicine, Beijing Hospital, Chinese Academy of Medical Sciences, Beijing, China

**Keywords:** chronic stress, atherosclerosis, inflammation, lipid metabolism, endothelial function, plaque stability

## Abstract

Atherosclerosis (AS) is a chronic vascular inflammatory disease, in which the lipid accumulation in the intima of the arteries shows yellow atheromatous appearance, which is the pathological basis of many diseases, such as coronary artery disease, peripheral artery disease and cerebrovascular disease. In recent years, it has become the main cause of death in the global aging society, which seriously endangers human health. As a result, research on AS is increasing. Lesions of atherosclerosis contain macrophages, T cells and other cells of the immune response, together with cholesterol that infiltrates from the blood. Recent studies have shown that chronic stress plays an important role in the occurrence and development of AS. From the etiology of disease, social, environmental and genetic factors jointly determine the occurrence of disease. Atherosclerotic cardio-cerebrovascular disease (ASCVD) is often caused by chronic stress (CS). If it cannot be effectively prevented, there will be biological changes in the body environment successively, and then the morphological changes of the corresponding organs. If the patient has a genetic predisposition and a combination of environmental factors triggers the pathogenesis, then chronic stress can eventually lead to AS. Therefore, this paper discusses the influence of chronic stress on AS in the aspects of inflammation, lipid metabolism, endothelial dysfunction, hemodynamics and blood pressure, plaque stability, autophagy, ferroptosis, and cholesterol efflux.

## Introduction

Atherosclerosis (AS) is considered as a non-specific inflammatory disease, mainly involving the intima and medial layer of the arterial wall, which is the pathological basis of various cardiovascular and cerebrovascular diseases (1, 2). Cardiovascular disease is still the leading cause of death worldwide, with an increasing prevalence in developing countries (3). In recent years, the rapid economic development in China has led to the change of lifestyle and the aggravation of population aging (4). The incidence and prevalence of chronic non-communicable diseases, such as hypertension, hyperlipidemia, diabetes, hyperuricemia, and chronic psychological stress, are increasing year by year, and the AS caused by these diseases is also becoming more and more serious (5–9). AS is the main cause of atherosclerotic cardiovascular disease (ASCVD), which is the leading cause of disability and death among urban and rural residents in China (10). In China, cardiovascular diseases account for about 45% of the deaths of the population, causing a serious medical burden and becoming a major public health problem (11). What's more, the incidence of ASCVD in China continues to rise. As is the main cause of ASCVD (12). The pathophysiological development of AS is closely related to the mutation and abnormal expression of genes, including fms-like tyrosine kinase-1 (Flt-1), tumor necrosis factor-α (TNF-α), apolipoprotein A-I (apo A-I), Vascular Endothelial Growth Factor (VEGF), and Angiogenin (ANG). Previous studies have shown that low expression of Flt-1 could predict the development of endothelial injury, which leads to the development of AS (13). In addition, the stronger the proliferative ability of endothelial progenitor cells (EPCs), the lower the vulnerability of vascular endothelium. Thus, TNF-α overexpression damages the vascular endothelium by disrupting the proliferation process of EPCs (14). The mutation of the anti-atherosclerosis gene, apo A-I, could accelerate the apoptosis of vascular endothelial cells by down-regulating the levels of endothelial nitric oxide synthase (eNOS) and heme oxygenase-1, and eventually lead to the formation of atherosclerotic plaque (15). The expression of VEGF and ANG could promote the regeneration of vascular endothelial cells (16, 17). Therefore, the abnormal expression of VEGF and ANG might play an important role in the occurrence and development of AS (18–20). The up-regulation of “VEGF and ANG” plays a significant role in the development of AS. Compared with the normal artery tissues, the expression of “VEGF and ANG” were higher in the AS tissues. The main biological function of ANG is to promote angiogenesis, which promotes plaque instability (21). VEGF is the strongest known factor promoting angiogenesis, which could promote endothelial cell mitosis and proliferation, increase vascular permeability and promote endothelial cell migration (22). Furthermore, VEGF could promote intimal hyperplasia and aggravate AS by promoting monocyte activation, adhesion, and migration and increasing permeability of endothelial cells (23). However, one of the important reasons for the current inability to effectively control the occurrence and recurrence of ASCVD is that the occurrence and progress of atherosclerotic stenosis and vulnerable plaques cannot be detected in time, dynamically monitored, and effectively controlled, which is also the main research field for ASCVD in China and abroad (24–26). So it is imperative to explore the risk factors for the occurrence and development of atherosclerosis for the early diagnosis and precise treatment of ASCVD (27).

Chronic stress induces changes in organisms that increase the risk of atherosclerotic diseases, including heart disease, stroke, and transient ischemic attack (8, 28). The report shows that stress increases the risk of cardiovascular disease (29). A large amount of evidence confirms that chronic stress plays a significant role in the occurrence and development of AS, but the specific mechanism is still unclear (30–33). The purpose of this paper is to provide a comprehensive review of studies on the effects of chronic stress in healthy individuals and patients with cardiovascular disease (CVD). This study focuses on the research progress of the relationship between chronic stress and AS in the aspects of inflammation, lipid metabolism, endothelial dysfunction, hemodynamics and blood pressure, plaque stability, autophagy, ferroptosis, and cholesterol efflux.

## Methods

**Figure 1:**
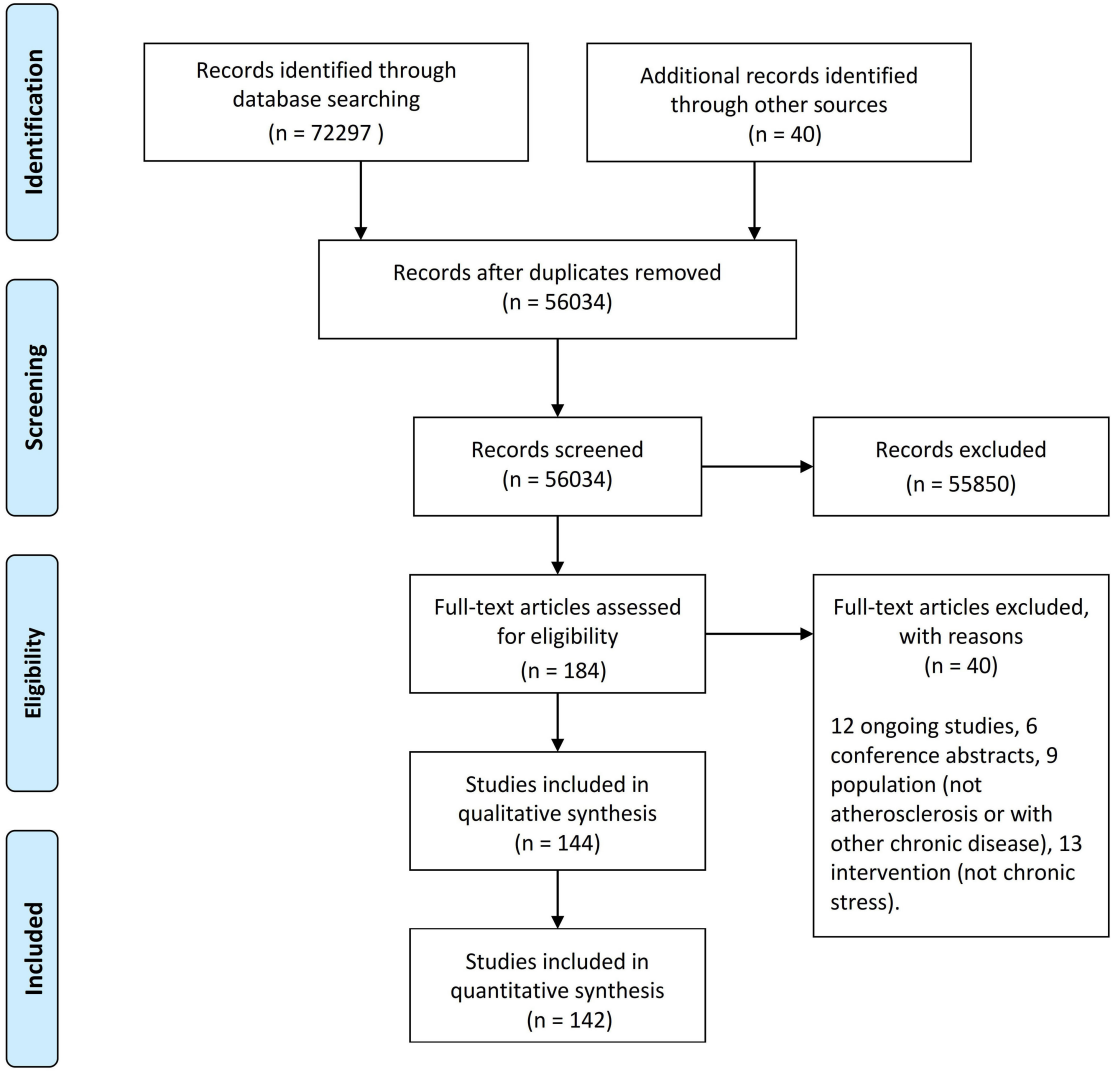
Flow diagram showing the procedure of searching the references in the databases.

Our systematic review is a new method of literature synthesis. It systematically and comprehensively collects the published and unpublished studies on a specific clinical problem, and uses the principles and methods of strict evaluation of clinical epidemiology to select the literatures that meet the quality standards for qualitative combination, so as to draw reliable comprehensive conclusions. This systematic review was conducted in accordance with the Preferred Reporting Items for Systematic Reviews statement guidelines. A protocol was developed prior to commencing this review on PROSPERO. The procedure of searching the references in the databases was manifested in the flow diagram (Figure 1; Table 1).

**Table 1:** Literature search tracking sheet.

**Date of search**	**Database**	**Years searched**	**Search terms**	**Strings of terms**	**#HITS**
20/05/21	PubMed, MEDLINE,MBASE, and Cochrane Library	2011–01/2021	Chronic stress	None used	52888
22/05/21	PubMed, MEDLINE,MBASE, and Cochrane Library	2011–01/2021	Chronic stress; atherosclerosis	(chronic stress[Title/Abstract]) AND (atherosclerosis[Title/Abstract])	182
23/05/21	PubMed, MEDLINE,MBASE, and Cochrane Library	2011–01/2021	Atherosclerosis; plaque stability	(Atherosclerosis[Title/Abstract]) AND (plaque stability[Title/Abstract])	655
24/05/21	PubMed, MEDLINE,MBASE, and Cochrane Library	2011–01/2021	Atherosclerosis; inflammation	(Atherosclerosis[Title/Abstract]) AND (inflammation[Title/Abstract])	12619
25/05/21	PubMed, MEDLINE,MBASE, and Cochrane Library	2011–01/2021	Atherosclerosis; lipid metabolism	(Atherosclerosis[Title/Abstract]) AND (lipid metabolism[Title/Abstract])	1706
25/05/21	PubMed, MEDLINE,MBASE, and Cochrane Library	2011–01/2021	Atherosclerosis; endothelial function	(Atherosclerosis[Title/Abstract]) AND (endothelial function[Title/Abstract])	1898
20/10/21	PubMed, MEDLINE,MBASE, and Cochrane Library	2011–10/2021	Atherosclerosis; autophagy	(Atherosclerosis[Title/Abstract]) AND (autophagy[Title/Abstract])	956
20/10/21	PubMed, MEDLINE,MBASE, and Cochrane Library	2011–10/2021	Atherosclerosis; ferroptosis	(Atherosclerosis[Title/Abstract]) AND (ferroptosis[Title/Abstract])	68
20/10/21	PubMed, MEDLINE,MBASE, and Cochrane Library	2011–10/2021	Atherosclerosis; cholesterol efflux	(Atherosclerosis[Title/Abstract]) AND (cholesterol efflux[Title/Abstract])	1325

### Search Strategy

This systematic review focused on the period 2011–2021. Main focus of this paper is on basic medical research about the AS and chronic stress. Researches included in this paper were screened by keyword searches in PubMed, MEDLINE, EMBASE, and Cochrane Library databases. These databases were searched using a combination of subject headings (such as MeSH) and filters (such as Time) when available. We reviewed references of included studies to identify pertinent studies. We imposed no language restriction. The keywords included “chronic stress,” “atherosclerosis,” “inflammation,” “lipid metabolism,” “endothelial function,” and “plaque stability.” And the strings of terms were “(chronic stress[Title/Abstract]) AND (atherosclerosis[Title/Abstract]),” “(Atherosclerosis[Title/Abstract]) AND (plaque stability[Title/Abstract]),” “(Atherosclerosis[Title/Abstract]) AND(inflammation[Title/Abstract]),” “(Atherosclerosis[Title/Abstract]) AND (lipidmetabolism[Title/Abstract]),” “(Atherosclerosis[Title/Abstract]) AND (endothelialfunction[Title/Abstract]),” “(Atherosclerosis[Title/Abstract]) AND (autophagy [Title/Abstract]),” “(Atherosclerosis[Title/Abstract]) AND (ferroptosis [Title/Abstract]),” “(Atherosclerosis[Title/Abstract]) AND (cholesterol efflux [Title/Abstract]).”

### Inclusion and Exclusion Criteria

Two reviewers independently assessed records identified from the search for eligibility. Any discrepancies were resolved by consensus. We included any studies referring to chronic stress and atherosclerosis. The researches mainly included the basic medical study with molecular exploration. Outcomes must be objectively measured “atherosclerosis.” We accepted 2011–2021 duration of intervention.

We excluded studies with confounding chronic conditions such as “ventricular remodeling, arrhythmia, Peripheral hemangitis.”

### Study Quality

Study quality was assessed by two reviewers based on the seven domains defined by the Cochrane Collaboration's tool for assessing risk of bias. Namely, (1) random sequence generation; (2) allocation concealment; (3) blinding of participants and personnel; (4) blinding of outcome assessment; (5) incomplete outcome data; (6) selective reporting; and (7) other biases, including baseline imbalance, early stopping and bias due to vested financial interest or academic bias.

Potential publication bias across studies was assessed using a funnel plot.

### Data Extraction

One author extracted all the data, and two authors reviewed the data for accuracy. The following data was collected: all papers about the association between “chronic stress” and “atherosclerosis.”

## Results and Discussion

### Chronic Stress Accelerating Atherosclerosis *via* Inflammation

Although the specific biological mechanisms by which chronic stress increases cardiovascular disease risk remain unclear (34). However, chronic low-grade inflammatory load appears as a possible link because chronic stress exacerbates this load and leads to early progression of atherosclerosis and thrombotic complications (35–37). Inflammation plays a key role in the overall atherosclerotic step, involving the accumulation of foam cells, the formation of fatty stripe tissue and fibrous plaques, the rupture of acute plaques, and the formation of thrombus (38–40). Persistence of inflammation is necessary for plaque development and instability, and plays a decisive role in the pathogenesis and progression of coronary artery disease (41–44). Animal experiments have shown that the levels of intercellular adhesion molecule-1 (ICAM-1), the reactant C-reactive protein (CRP) in the acute phase, and the pro-inflammatory cytokine are significantly increased in apolipoprotein E (ApoE) knockout mice preconditioned by chronic stress (45, 46). Plenty of evidence shows that chronic stress could activate inflammation in the brain and surrounding areas (47, 48). Some researchers believe that stress might activate the Sympathetic Nervous System (SNS) to release noradrenaline (NE) and Nerve Peptide Y (NPY), and these two stress hormones further promote the phosphorylation of mitogen activated protein kinases (MAPKs) or the release of High Mobility Group Box 1 (HMGB1), thereby inducing systemic inflammation to accelerate the development of CVD (49). Chronic stress alters the dynamic balance of the sympathetic and vagal nervous systems. Decrease of vagal tone could promote inflammation. It has been found that chronic stress could enhance the activity of dipeptidyl peptidase-4 (DPP4) in plasma and reduce plasma glucagon-like peptide-1 (GLP-1) and adiponectin (APN) concentrations, thus promoting the development of inflammation (50–52). However, whether it is possible to reduce the promoting effect of chronic stress on atherosclerosis through the targeted inhibition of some cellular inflammatory factors remains to be further studied.

It has been proved that chronic stress and its related diseases anxiety and depression interact with inflammatory response (53). IL-6 is an important inflammatory factor, and its changes represent the body's defense response to chronic stress and help the body adapt to the environment (54). And IL-6 is a kind of polypeptide cytokines with immunomodulatory effects, mainly produced by mononuclear macrophages and T lymphocytes. In the central nervous system, both neurons and glial cells produce this factor (55). Study has shown that IL-6 is involved in the occurrence and development of atherosclerosis in hypertensive patients, and the size of cerebral infarction is positively correlated with the level of serum IL-6 (56). At the same time, IL-6 might promote the progression of atherosclerosis. Studies have shown that IL-6, a pro-inflammatory factor, is elevated in the serum of patients with chronic stress (57). Serum IL-6 increased significantly after chronic stress, and the increase was more obvious in the high-fat diet group. Cortisol acts as an anti-inflammatory, and as IL-6 levels rise in the body, so do cortisol levels. Studies have shown that chronic stress can promote the development of AS through high levels of cortisol mediated by IL-6 (58). IL-6 could promote platelet activation, accelerate the coagulation process, cause endothelial and smooth muscle cell necrosis, and accelerate the formation of AS. IL-6 could damage the vascular endothelium and interfere with uptake of low-density lipoprotein (LDL) by macrophages, resulting in lipid accumulation in the vascular wall and leading to AS (59).

### Disorder of Lipid Metabolism in Atherosclerosis Under Chronic Stress

Studies have shown that chronic stress-induced hyperlipidemia and oxidative damage can contribute to the development of atherosclerosis (60). Although atherosclerosis is a chronic inflammatory disease, currently, more and more evidence manifested that atherosclerosis is a complex systematic pathology, and hyperlipidemia is a major risk factor for changes in intimal and media thickness during atherosclerosis (41). Experiments have found that compared with the control group, the high concentrations of serum total cholesterol, triglyceride, low density lipoprotein cholesterol (LDLC), and very low density lipoprotein cholesterol (VLDLC) could increase the atherosclerosis index in the chronic stress group, while the concentration of high density lipoprotein cholesterol did not change significantly (61–65). Chronic stress caused by long-term social pressure leads to obesity to some extent. Obesity is the result of excessive accumulation of fat (66). Scientific studies have shown that obesity can increase the incidence of cardiovascular and cerebrovascular diseases (67, 68). However, the accumulation of subcutaneous fat was not associated with an increased risk of cardiovascular disease. One study found that chronic stress promoted the accumulation of visceral fat, which in turn led to atherosclerosis and cardiovascular events, rather than the accumulation of subcutaneous fat (69). The chronic stress might stimulate the production of glucocorticoid, which can promote visceral obesity, and accompanied by a series of metabolic disorders, including dyslipidemia, impaired glucose tolerance and insulin resistance, unstable or elevated blood pressure (70–73). These factors will be harmful to the arteries, and promote the development of atherosclerosis (67, 74). Other studies have found that Neuropeptide Y (NPY) is a mediator of vascular lipid metabolism disorder under chronic stress and a risk factor for stress-induced lipid metabolic syndrome and atherosclerosis (75–78). Understanding how neuropeptide Y and its homologous receptors regulate lipid metabolism may provide new ideas for the study of the mechanism and treatment of atherosclerosis (79, 80). A large number of studies have shown that hyperlipidemia, induced by chronic stress, is closely related to atherosclerosis (60, 81–83). Therefore, the understanding of lipid metabolism under stress state has important guiding significance for the study of the relationship between chronic stress and atherosclerosis.

### Effect of Chronic Stress on Endothelial Dysfunction in Atherosclerosis

Studies have shown that stress is a risk factor for cardiovascular disease (CVD) (84–86). However, the underlying mechanism is not clear. Studies have shown that mental stress activates the sympathetic nervous system (87), which might cause a range of adverse cardiovascular effects, including increased blood pressure, increased heart rate, and endothelial dysfunction. The endothelial dysfunction represent an important link between chronic stress and cardiovascular disease (CVD) risk (46, 88). Recent data from human and animal stress model studies highlight the critical role of endothelial dysfunction in stress-induced cardiovascular disease (89). It was found that under chronic stress, thoracic aortic rings exhibited high sensitivity to vasoconstrictors by inhibiting nitric oxide synthase activity or removing endothelial cells (90–92). Chronic stress could reduce NO production and induce physiological and biological changes of blood vessels, leading to endothelial dysfunction and the progression of atherosclerotic plaques (93, 94). One study examined the effect of vascular endothelial dysfunction on subclinical atherosclerotic plaques by measuring arterial elasticity by observing changes in the percentage of intima-media. The results showed that the loss of endothelial cells could affect the percentage of intima-media and induce atherosclerosis. It has also been found that poor vascular endothelial function will increase the incidence of atherosclerosis (95, 96). Endothelial dysfunction is an important cause of atherosclerosis. Stress can directly inhibit the vasodilator function of endothelial cells. Patients with long-term chronic psychological stress may develop impaired vascular endothelial function. Maintaining homeostasis is a new way to prevent and treat atherosclerosis.

### Variation of Hemodynamics and Blood Pressure Under Chronic Stress

Chronic stress is associated with increased cardiovascular risk, including increased incidence of atherosclerosis, myocardial ischemia, coronary heart disease, and death. The association between stress and cardiovascular dysfunction represents an important node for therapeutic interventions for cardiovascular disease, especially in the aging population, where hypertension is a well-known risk factor (97). Chronic stress plays a very important role in the development of hypertension, and its mechanisms are known to involve long-term abnormal neurological and endocrine activity, such as significantly elevated levels of corticosteroids, cortisol, epinephrine, norepinephrine, and angiotensin. Initially, the sympathetic nerve-adrenal medulla system is an important factor in the development of hypertension. Under chronic stress, plasma adrenaline, norepinephrine, and dopamine increase rapidly (98). It is now clear that in hypertension, the sympathetic nervous system activity is increased, and sympathetic excitation causes small arteriovenous contractions, leading to an increase in diastolic/systolic blood pressure (99–101). Catecholamine is an important humoral factor in the sympathetic adrenal myeloid system, which can cause constriction of peripheral blood vessels and increase diastolic pressure. The renin-angiotensin-aldosterone system also plays an important role in chronic stress by inducing increased angiotensin levels, regulating catecholamine secretion, and increasing blood pressure (102–105). Sympathetic excitation is known to increase angiotensin II production by stimulating proximal cells and beta receptors in local tissues to promote renin secretion. Finally, on the hypothalamic-pituitary-adrenal axis (106, 107), chronic psychological stress stimulates the secretion of corticosteroid releasinghormone (CRH) and vasopressin (AVP) in the hypothalamus, which promotes the secretion of corticotropic hormone. Glucocorticoids are important factors in maintaining the normal response of the circulatory system to catecholamines. Glucocorticoid deficiency was associated with significantly reduced response, decreased myocardial contractibility, decreased output, and decreased blood pressure (108). In addition, endothelin (ET) was also an important factor regulating cardiovascular function, and plays an important role in maintaining vascular tension and cardiovascular system homeostasis. As endodermal vascular active factors, endothelin has the strongest and most lasting effect among the endogenous vasoconstrictor peptides. The endothelium could contract vessels and promote endothelial cell proliferation by releasing endothelin. Hypertension and diabetes could lead to endothelial dysfunction and promote release of endothelin (109). The levels of endothelin in patients with diabetes and coronary heart disease were higher than those in control group. The level of endothelin increased significantly in diabetic patients with coronary heart disease. These results demonstrate that endothelin is a good response to vascular endothelial disease regardless of the primary etiology. One study suggests that plasma endothelin levels in atherosclerotic patients are proportional to the severity of atherosclerotic vascular lesions. The more damaged vessels, the higher the endothelin level (110). Endothelin might be an independent risk factor for atherosclerosis. Endothelin causes coronary artery dysfunction, promotes coronary artery wall remodeling, platelet activation, and aggregation (111).

### Reduced Plaque Stability by Chronic Stress

Chronic stress could reduce the intimal mediators of atherosclerosis and accelerate plaque instability by promoting apoptosis and neovascularization (28). In our current study, chronic stress increased plaque vulnerability, characterized by thinning of the fibrous cap, larger lipid nuclei, increased macrophages and neovascularization, but fewer smooth muscle cells and elastic fibers (112–114). Thus, chronic stress may not induce larger plaque areas, but rather lead to advanced atherosclerotic lesions. So, how does chronic stress affect the stability of atherosclerotic plaque? Levels of inflammation and oxidative stress, which can be exacerbated by chronic stress, have been shown to be associated with atherosclerotic plaque instability (115, 116).

### The Effect of Chronic Stress on Atherosclerosis *via* Autophagy

Autophagy is a self-protective cellular catabolic pathway involved in protein and organelle degradation (117, 118). Autophagy plays an important role in inhibiting inflammation and apoptosis, and in promoting efferocytosis and cholesterol efflux, and in maintaining cellular metabolic homeostasis. Autophagy is related to oxidative stress, inflammation, and foam cell formation, further promoting atherosclerosis. Therefore, autophagic homeostasis is essential for the development and outcome of atherosclerosis (119). Atherosclerotic lesions are continuously challenged by stressful insults such as DNA damaging molecules, ROS, oxidized lipids, inflammatory cytokines, hypoxia, etc. and will respond in three different ways: either fight (autophagy), adapt (senescence), or die (apoptosis/necrosis). All the three pathways are interconnected and negatively control each other. Atherosclerosis is the progressive buildup of plaque in the arterial wall ultimately resulting in rupture and thrombosis manifesting (120). Moderate activation of autophagy prevents macrophages and vascular smooth muscle cells (VSMCs) from forming foam cells and preventing the progression of atherosclerotic plaques (121, 122). Stimulation of autophagy suppresses vascular smooth muscle cell senescence, whereas inhibition of autophagy promotes it (123). Autophagy is an evolutionarily conserved process in eukaryotes that processes the turnover of intracellular substances. In patients, excessive autophagy activation leads to cell death, plaque instability, or even plaque rupture (119, 124). Abnormal autophagy regulation may lead to atherosclerosis (125).

### The Relationship Between Atherosclerosis and Ferroptosis

Ferroptosis is a newly identified form of regulated cell death characterized by the iron-dependent accumulation of lipid hydroperoxides to lethal levels (126), this type of cell death was found to have molecular characteristics distinct from other forms of regulated cell death (127), which exhibits distinct features from apoptosis, necrosis and autophagy in morphology, biochemistry, and genetics (128, 129). Ferroptosis is a type of autophagy-dependent cell death (130). Emerging mechanisms of ferroptosis is related to disease (131). Ferroptosis is closely related to atherosclerosis, and might occur during the initiation and development of AS (129). Apoptosis, necrosis and autophagy-dependent cell death are the three major types of cell death. Traditionally, necrosis is thought as a passive and unregulated form of cell death. However, certain necrosis can also occur in a highly regulated manner, referring to regulated necrosis. Depending on the signaling pathways, regulated necrosis can be further classified as necroptosis, pyroptosis, ferroptosis, parthanatos, and CypD-mediated necrosis. endothelial progenitor cell (EPC)-EVs transferred miR-199a-3p to inhibit Sp1 Transcription Factor (SP1), thus repressing ferroptosis of endothelial cells and retarding the occurrence of AS (132). Inhibition of ferroptosis could alleviate AS through attenuating lipid peroxidation and endothelial dysfunction in AECs (129, 133). Therefore, ferroptosis as a central gene in human coronary atherosclerosis (134).

### Aggregating Atherosclerosis *via* Cholesterol Efflux Under the Chronic Stress

Cholesterol is an important lipid for maintaining cell membrane fluidity and generation of various hormones and bile acids. Thus, it is critical to maintain cholesterol homeostasis including absorption, trafficking, biosynthesis, and efflux. Dysregulation of cholesterol homeostasis may lead to human disorders (135). The phenomena of lipid accumulation, inflammation, oxidative stress, hypoxia, and insulin resistance commonly associated with AS lesions can regulate the expression of cholesterol transporter, and then regulate intracellular cholesterol efflux, affecting the occurrence, and development of As. Cholesterol efflux is a key step in cholesterol reverse transport (136). The reverse cholesterol transport, a process that removes excess cholesterol from peripheral tissues/cells including macrophages to circulating HDL, is one of the main mechanisms responsible for anti-atherogenic properties of HDL. Reverse cholesterol transport (RCT) may counteract the pathogenic events leading to the formation and development of atheroma, by promoting the high-density lipoprotein (HDL)-mediated removal of cholesterol from the artery wall (137, 138). The key proteins of reverse cholesterol transport-ATP-binding cassette transporters A1 (ABCA1) and G1 (ABCG1)-mediate the cholesterol efflux from macrophages and prevent their transformation into foam cells (139). The formation of foam cells is a typical pathological feature of early atherosclerosis, the imbalance of cholesterol metabolism homeostasis of macrophages runs through the whole process of foam cell formation.

Atherosclerosis is characterized by significant aggregation of macrophage foam cells in atherosclerotic plaques and associated pro-inflammatory responses in pathological cells. Results from animal and human studies suggest that in these cells, especially in diseased macrophages, dyshomeostasis plays a key role in the pro-inflammatory response. The cholesterol efflux pathway also inhibits the accumulation of cholesterol esters in macrophages, namely the formation of macrophage foam cells (140). Cholesterol efflux is a key link in regulating the cholesterol dynamic balance of macrophages, which is of great significance in reducing intracellular cholesterol accumulation, preventing the formation of foam cells and the occurrence of As. Cholesterol efflux pathways exert anti-inflammatory and anti-atherogenic effects by suppressing proliferation of hematopoietic stem and progenitor cells, and inflammation and inflammasome activation in macrophages. Therefore, atherosclerosis can be prevented by promoting cholesterol efflux from macrophages (141, 142).

**Figure 2:**
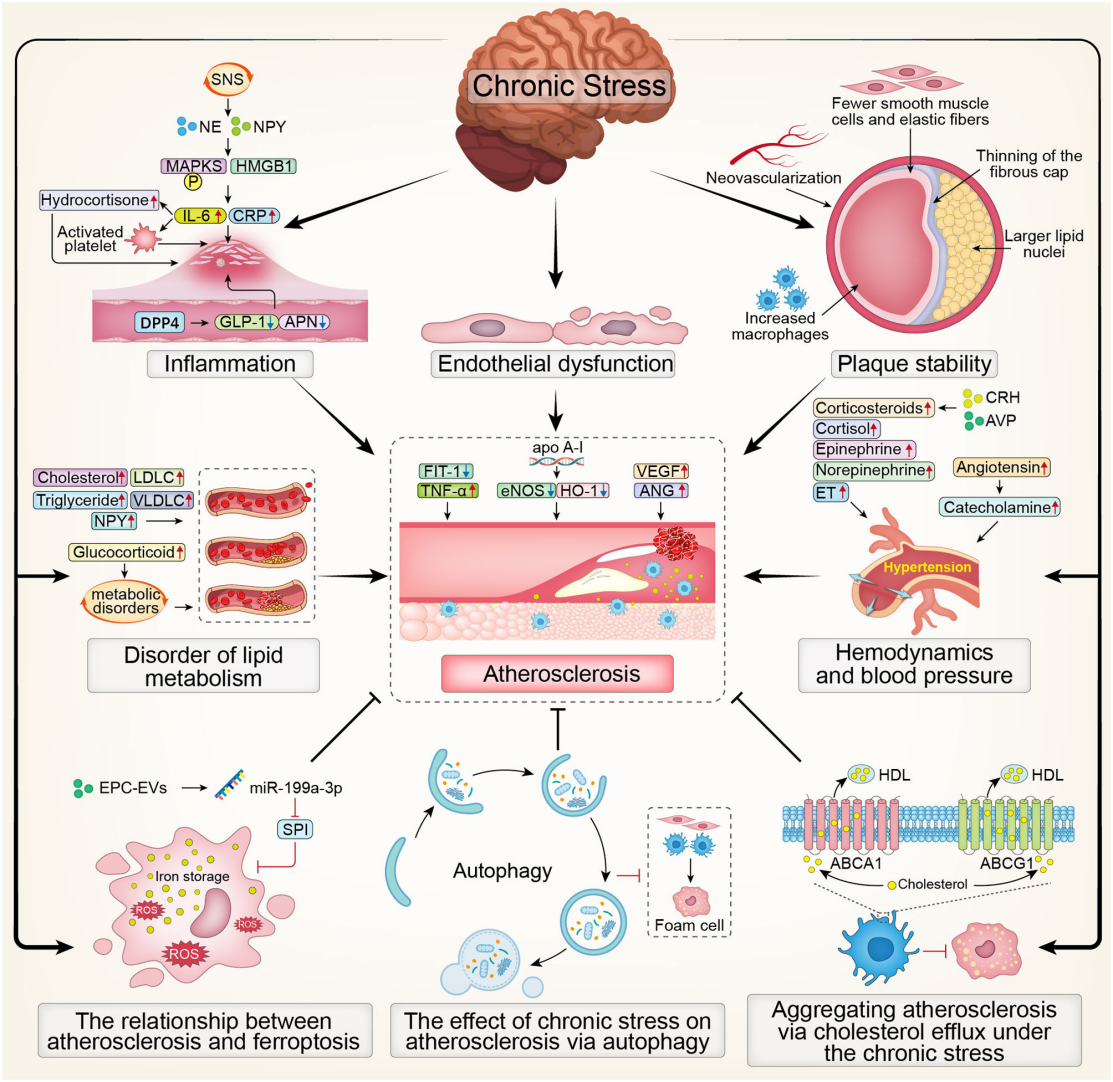
The overview map presenting the effect of chronic stress on atherosclerosis. SNS, Sympathetic Nervous System; NE, noradrenaline; NPY, Nerve Peptide Y; MAPKS, mitogen activated protein kinases; HMGB1, High Mobility Group Box 1; CRP, C-reactive protein; IL-6, interleukin; DPP4, dipeptidyl peptidase-4; GLP-1, glucagon-like peptide-1; APN, adiponectin; LDLC, low density lipoprotein cholesterol; VLDLC, very low density lipoprotein cholesterol; Flt-1, fms-like tyrosine kinase-1; TNF-α, tumor necrosis factor-α; eNOS, endothelial nitric oxide synthase; HO-1, Hemeoxygenase-1; VEGF, Vascular Endothelial Growth Factor; ANG, Angiogenin; CRH, corticosteroid releasinghormone; AVP, vasopressin; ET, endothelin; ROS, Reactive oxygen species; EPC, endothelial progenitor cell; SP1, Sp1 Transcription Factor; HDL, high-density lipoprotein; ABCA1, ATP-binding cassette transporters A1; ABCG1, ATP-binding cassette transporters G1.

In summary, the overview map presented the effect of chronic stress on atherosclerosis (Figure 2).

## Conclusion

Chronic stress is the cause of atherosclerotic cardiovascular and cerebrovascular diseases. If it cannot be effectively prevented, biological changes in the body environment will occur successively, such as inflammation, lipid metabolism, endothelial function, hemodynamics and other changes, and then morphologic changes of the corresponding organs will appear. If the patient has a genetic predisposition, and at the same time the environmental factors work together to activate the pathogenic mechanism, then the chronic stress factors will eventually lead to the development of atherosclerotic cardiovascular disease.

## Data Availability Statement

The original contributions presented in the study are included in the article/supplementary material, further inquiries can be directed to the corresponding author/s.

## Author Contributions

L-bM was major contributor in writing and was involved in critically revising manuscript for important intellectual content. TG and D-pL made substantial contributions to research conception and designed the draft of the research process. YL and Y-mZ were major contributors in submitting the manuscript and they gave the technical support in the methods. All authors read and approved the final manuscript.

## Funding

The present study was funded by the National Key R&D Program of China (Grant Nos. 2020YFC2003000 and 2020YFC2003001), Chinese Academy of Medical Sciences, CAMS Innovation Fund for Medical Sciences (Grant No. 2018-I2M-1-002), and National Natural Science Foundation of China (Grant Nos. 31271097 and 51672030).

## Conflict of Interest

The authors declare that the research was conducted in the absence of any commercial or financial relationships that could be construed as a potential conflict of interest.

## Publisher's Note

All claims expressed in this article are solely those of the authors and do not necessarily represent those of their affiliated organizations, or those of the publisher, the editors and the reviewers. Any product that may be evaluated in this article, or claim that may be made by its manufacturer, is not guaranteed or endorsed by the publisher.

## Abbreviations

AS: Atherosclerosis
ASCVD: Atherosclerotic cardio-cerebrovascular disease
Flt-1: fms-like tyrosine kinase-1
TNF-α: tumor necrosis factor-α
apo A-I: apolipoprotein A-I
VEGF: Vascular Endothelial Growth Factor
ANG: Angiogenin
EPCs: endothelial progenitor cells
eNOS: endothelial nitric oxide synthase
CVD: cardiovascular disease
ICAM-1: intercellular adhesion molecule-1
CRP: C-reactive protein
ApoE: apolipoprotein E
SNS: Sympathetic Nervous System
NE: noradrenaline
NPY: Nerve Peptide Y
ET: endothelin
MAPKs: mitogen activated protein kinases
HMGB1: High Mobility Group Box 1
DPP4: dipeptidyl peptidase-4
GLP-1: glucagon-like peptide-1
APN: adiponectin
LDLC: low density lipoprotein cholesterol
VLDLC: very low density lipoprotein cholesterol
NPY: Neuropeptide Y
CRH: corticosteroid releasinghormone
AVP: vasopressin
RCT: Reverse cholesterol transport
HDL: high-density lipoprotein
ROS: Reactive oxygen species
EPC: endothelial progenitor cell
SP1: Sp1 Transcription Factor
HDL: high-density lipoprotein
ABCA1: ATP-binding cassette transporters A1
ABCG1: ATP-binding cassette transporters G1.
